# Feasibility and Acceptability of a Community-Based Health Literacy Program for Individuals With Prehypertension

**DOI:** 10.1177/01939459261469267

**Published:** 2026-08-01

**Authors:** Pataporn Bawornthip, Jo Mcdonall, Decha Tamdee, Andrea Driscoll, Anastasia Hutchinson

**Affiliations:** 1 School of Nursing and Midwifery, Faculty of Health, Deakin University, Geelong, Australia; 2 Centre for Quality and Patient Safety Research, Institute for Health Transformation, Faculty of Health, Deakin University, Geelong, Australia; 3 Faculty of Nursing, Chiang Mai University, Chiang Mai, Thailand; 4 School of Nursing and Midwifery, La Trobe University, Melbourne, Australia; 5 Centre for Quality and Patient Safety Research, Institute for Health Transformation, Faculty of Health, Deakin University–Monash Health Partnership, Geelong, Australia; 6 Centre for Quality and Patient Safety Research, Institute for Health Transformation, Faculty of Health, Deakin University–Epworth HealthCare Partnership, Geelong, Australia

**Keywords:** health literacy, health behaviors, blood pressure, prehypertension, community-based intervention, feasibility

## Abstract

**Background:**

Engaging end users through co-design is essential for developing effective and sustainable hypertension prevention interventions, while rigorous evaluation helps identify and address barriers to enhance acceptability and impact.

**Methods:**

A feasibility study was conducted using a convergent mixed-methods design. A convenience sample participated in a community-based Health Literacy Program. Instruments included the Thai Hypertensive Health Literacy and Health Behaviors Questionnaire (Thai HLHBQ), the Information and Support for Health Action Questionnaire (ISHA-Q), a hypertension knowledge test, a satisfaction survey, and an interview guide. Data were analyzed using paired *t*-tests, Wilcoxon signed-rank tests, and repeated measures ANOVA.

**Results:**

The sample comprised ten adults with prehypertension, one nurse practitioner, and one public health officer. The attendance rate of participants with prehypertension during the 6-week period was 100%. Participants completed a baseline assessment at Week 0. The intervention was delivered over a 5-week period (Weeks 1-5), and post-intervention assessments were conducted at Week 6. Post-intervention, significant improvements were observed in knowledge, health literacy, and blood pressure readings. Knowledge increased from 6.8 to 9.3/10 (Hedges’ *g* = 2.65, *P* < .001). Health literacy scores increased by 10 points (HLHBQ; Hedges’ *g* = 1.05, *P* = .005) and 32.5 points (ISHA-Q; *r* = 0.66, *P* = .037). Systolic blood pressure decreased from 129.3 to 123.9 mmHg (η^2^_p_ = 0.43, *P* < .004) and diastolic from 83.9 to 79.9 mmHg (η^2^_p_ = 0.41, *P* < .001).

**Conclusion:**

The results provide evidence that the intervention was both feasible and acceptable for implementation in a rural Thai community.

## Introduction

Empowering and engaging individuals, families, and communities enables them to make informed health decisions by co-designing healthy interventions, partnering in care delivery, and shaping public health policy.^1-3^ The Sustainable Development Goals (SDGs) for non-communicable diseases (NCDs) emphasize the importance of health literacy in enabling individuals, communities, and organizations to understand, protect, and promote health.^4^ Health professionals should prioritize the prevention, early detection, and treatment of NCDs through inclusive, equitable, and locally tailored strategies, ensuring no one is left behind.^1,4,5^

Health literacy is crucial for reducing health inequities and managing NCDs by enabling individuals to access, understand, and use health information to make informed decisions in daily life.^6^ Health promotion and health literacy are complementary concepts that can contribute to improved health outcomes.^6^ Health education is essential for improving health outcomes, increasing knowledge, and enhancing prevention and self-care management practices among individuals with chronic diseases, especially hypertension.^7-9^ Health literacy plays a key role in empowering sustainable healthcare by integrating it into educational sessions.^10^ Previous systematic reviews have shown that a higher level of health literacy is associated with improved health outcomes—such as blood pressure control, increased knowledge of self-management strategies, better medication adherence, and enhanced health-related quality of life in individuals with hypertension.^11-13^

The Optimizing Health Literacy and Access (Ophelia) process is a systematic approach used to plan and implement National Health Literacy Demonstration Projects for the prevention and control of NCDs.^5^ The process consists of 3 phases and 8 steps, focusing on the development, implementation, and evaluation of tailored health literacy interventions.^5^ This study was underpinned by the Ophelia framework, which guided the development, testing, and refinement of the intervention in terms of both process and materials.

The ultimate goal of preventing and treating elevated blood pressure and hypertension is to reduce cardiovascular disease, improve quality of life, and prevent premature death.^14^ A systematic review of risk factors for hypertension in Southeast Asian populations found that sociodemographic factors, including sex, ethnicity, education, health literacy, and socioeconomic status, were associated with hypertension.^15^ Moreover, a high body mass index and increased waist circumference were also crucial determinants of hypertension.^15^ According to the European Society of Cardiology and the Thai Hypertension Society, updated guidelines strongly recommend lifestyle changes to prevent and manage elevated blood pressure. These include engaging in moderate-intensity aerobic exercise, reducing body mass index and waist circumference, and adopting a healthy and balanced diet.^14,16^ A previous scoping review examining hypertension prevention among Thai individuals with prehypertension revealed that existing interventions primarily targeted blood pressure monitoring, knowledge enhancement, behavior change, social support, and counseling.^17^ These interventions largely focused on promoting preventive actions, nutrition, and raising disease awareness.^17^ Systematic reviews and meta-analyses of exercise and dietary interventions found that endurance exercise, a healthy diet, sodium restriction, and increased potassium intake were feasible and effective in Southeast Asian countries.^18,19^

However, none of these interventions were developed through co-design with the end users. The community-based Health Literacy Program for individuals with prehypertension (HeLP-PH) was co-designed and developed by healthcare professionals, community leaders, family members, and consumers, including individuals with prehypertension and hypertension. This intervention was guided by the Ophelia framework.^5^ The process involved surveying health literacy and health behaviors, and conducting a situation analysis, followed by designing and developing a health literacy intervention to improve health literacy and blood pressure control.

## Study Aim

We sought to evaluate the feasibility and acceptability of a co-designed, community-based HeLP-PH.

## Methods

### Design and Setting

This study employed a convergent mixed-methods design to evaluate the feasibility and acceptability of a co-designed community-based intervention to improve blood pressure control. The study was part of a research project conducted in primary health care setting located in Chiang Mai province in rural northern Thailand, based on the Optimizing Health Literacy and Access (Ophelia) framework.^5^ The Good Reporting of A Mixed-Methods Study checklist was used to instruct this report.

### Intervention Approach

The community-based HeLP-PH was delivered over 5 weeks (Supplemental Table S1). The intervention included: (1) a community-based educational project that involved community members sharing real-life experiences and cooking with low-sodium condiments; (2) a blood pressure monitoring system that provided equipment and a handbook with step-by-step instructions for measuring blood pressure and related health information; and (3) public relations activities delivered via a local broadcasting tower and the digital communication application “Line,” which integrated rural community communication networks with digital technology (Supplemental Figure S1).

### Study Outcomes

Feasibility outcomes were pre-specified and included recruitment rate, retention rate, intervention attendance, and acceptability. Recruitment and retention rates were calculated as the proportions of eligible participants who enrolled in and completed the study, respectively. Attendance was assessed using session participation records, while acceptability was evaluated through participant satisfaction with the intervention program at follow-up and semi-structured interviews.

Secondary outcomes included: change in systolic and diastolic blood pressure between baseline, the 5-week period, and the Week-6 follow-up; change in knowledge of risk factors for hypertension and importance of blood pressure control; uptake of health-promoting behaviors; and change in health literacy, waist circumference, and body mass index between baseline and follow-up.

### Sample Size

A purposive sample of health care workers (n = 2) was enrolled to conduct in-depth interviews. A convenience sample of participants (n = 10) was enrolled in a community-based HeLP-PH. This small-scale setup study was conducted^5^ to evaluate the feasibility and acceptability of the proposed intervention, and to inform design and development of a future definitive study using a randomized controlled trial approach. Each of the 10 participants provided 6 repeated measures of blood pressure (including baseline measurements and 5 weeks of follow-up), providing a sample size of 50 measurements for the primary outcome variable, which is sufficient for a proof-of-concept pilot study.^20^

### Inclusion and Exclusion Criteria

Inclusion criteria comprised adults aged 20 years and older who were diagnosed with prehypertension (defined as having a systolic blood pressure of 130-139 mmHg or a diastolic blood pressure of 85-89 mmHg). Also included were participants who could read, write, and communicate in the Thai language, and either use a smartphone themselves or had a family member with a smartphone. Adults diagnosed with hypertension were excluded.

### Data Collection and Measures

Individuals with prehypertension who met the inclusion criteria were invited to participate and asked to sign an informed consent form. Data collection was conducted between November and December 2024. At baseline, sociodemographic information and clinical outcomes—including blood pressure, waist circumference, body mass index, health literacy, and health behaviors—were collected. Clinical outcomes were measured weekly for 4 weeks, while health literacy and health behaviors were reassessed 1-week following completion of the intervention.

#### Thai Hypertensive Health Literacy and Health Behaviors Questionnaire

This questionnaire was validated, and confirmatory factor analysis (CFA) demonstrated an adequate fit of the measurement model.^21^ This 16-item questionnaire was recorded with a 5-point Likert scale: never = 1, very difficult = 2, hard to do = 3, easy to do = 4, very easy to do = 5. Responses to the 16 items were totaled, and total health literacy scores were categorized as: poor (score of 16-47), fair (score of 48-55), good (score of 56-63), or very good (score of 64-80).

The health behavior questionnaire measured adherence to diet and exercise recommendations and consisted of 8 items, using a 5-level Likert scale that assesses the frequency with which these activities are performed each week. Dietary and exercise behaviors were scored and classified as: not good (scores 6-17), fair (18-20), good (21-23), and very good (24-30); exercise behavior was classified as poor (scores 2-5), fair (6), good (7), and very good (8-10).

#### Information and Support for Health Action Questionnaire

This questionnaire demonstrated construct validity, and CFA indicated an adequate fit for the measurement model (Supplemental Table S2). It consisted of 67 items across 16 scales, with responses scored on a 0 to 10 scale ranging from “strongly disagree” to “strongly agree,” resulting in a total possible score of 670 points.

#### Knowledge of Hypertension and Health Behaviors Test

The knowledge of hypertension and health behaviors test was measured before commencement and immediately after completing the community-based educational project. The test consisted of 10 items, each with 4 answer choices, and the total score was 10.

#### Satisfaction Survey

The satisfaction survey was conducted to evaluate 4 domains of trainer performance: knowledge and understanding, application of knowledge, and project implementation. This 18-item questionnaire used a 5-point Likert scale: 1 = Improve, 2 = Fair, 3 = Neutral, 4 = Good, and 5 = Very Good. The mean overall score was classified as follows: Improve (1.00-1.50), Fair (1.51-2.50), Neutral (2.51-3.50), Good (3.51-4.50), and Excellent (4.51-5.00).

#### Interview Guide

We used a semi-structured interview guide for conducting in-depth interviews, consisting of 5 questions, and focus group interviews, consisting of 17 questions.

### Data Analysis

#### Statistical Analyses

The satisfaction survey conducted after the education session was analyzed using descriptive statistics (mean, standard deviation, and percentage). The data on health literacy measured by the Information and Support for Health Action Questionnaire (ISHA-Q) were not normally distributed; therefore, a Wilcoxon signed-rank test was conducted. In contrast, the data on knowledge of hypertension and health behaviors, as well as health literacy and health behaviors measured by the Thai Hypertensive Health Literacy and Health Behaviors Questionnaire (Thai HLHBQ), were normally distributed. Therefore, paired *t*-tests were used to test mean differences. The data distribution was tested using the Shapiro-Wilk test and visualized with a Q-Q plot.^22^

Repeated measures ANOVA was used to analyze systolic blood pressure, diastolic blood pressure, waist circumference, and body mass index. Mauchly’s test of sphericity was significant for systolic blood pressure, so the assumption of sphericity was met. However, for diastolic blood pressure, waist circumference, and body mass index, Mauchly’s test was non-significant, and the Greenhouse–Geisser correction was applied.^23^

IBM SPSS version 30.0 (IBM Corp., Armonk, NY, USA) was used for all data analyses.

#### Thematic Analysis

One focus group and two in-depth interviews were analyzed using thematic analysis.^24^ The thematic analysis consisted of 6 steps: familiarization with the dataset, generation of codes, development of initial themes, reviewing and refining themes, defining and naming themes, and reporting the findings.^24^

## Results

A total of 12 participants were included in the study, including 10 individuals with prehypertension and 2 healthcare workers (HCWs) from a primary health care site. The mean age of individuals with prehypertension was 50.9 years (±10.4), and 90% of participants were female. Most participants were married (80%) and employed (90%) (Table 1). Both HCWs were female, with a mean age of 35 years (±1.4). The recruitment rate was 100% and the attendance rate of participants with prehypertension during the 6-week period was 100%.

**Table 1.** Characteristics of Participants (n = 10).

Characteristics	n	%
Sex		
Female	9	90
Age (years) Mean 50.9, SD 10.4, Range 30-64		
Age group		
30-40	2	20
41-50	1	10
51-60	6	60
61 and over	1	10
Marital status		
Married	8	80
Single	1	10
Widowed	1	10
Education		
Primary education	3	30
High school	4	40
Bachelor’s degree or over	3	30
Employment		
Employed	9	90

### Feasibility and Acceptability of the Health Literacy Intervention

Nine themes were identified from the in-depth interviews with HCWs and focus group discussions with individuals with prehypertension (Table 2). The health education program was acceptable and motivated behavior change. Three participants reported that the training was acceptable, highlighting its practicality for everyday application and accessibility, including a suitable schedule and convenient transportation. However, some participants recommended reducing the group size to enhance participation and concentration. One participant suggested providing rewards to motivate attendance and engagement in the training. The BP wellness kit was considered appropriate and useful. Participants reported that the handbook was helpful and easy to read with a big font size and colors, particularly for older adults.

**Table 2.** Summary of Feasibility and Acceptability of the Health Literacy Intervention.

Health literacy intervention	Engagement in the intervention	Feasibility and acceptability as perceived by participants	
		Theme	Representative of quotes
1. A community-based educational project on hypertension	All participants in the intervention group (n = 10) attended the education session.	1. Education was acceptable and motivated behavior change	*“The training included both theoretical and practical components, and involved community members to encourage behaviour change, which added credibility and motivated individuals with prehypertension to take action”* (HCW2) “*A half-day training session was appropriate. A full-day session would be too exhausting for participants. The location at the health center was also suitable, though a few had minor issues with knee pain. Overall, everyone seemed happy”* (HCW1) *“Training should be conducted every three months to provide refreshers and basic health checks. A half-day session (9 a.m. to noon) is ideal, as full-day sessions are too demanding given participants’ other responsibilities”* (P1)
		2. Recommendations for the next phase of development	“*I would prefer if the training were divided into learning stations, such as Station 1: Self-awareness and identifying risk factors, Station 2: Health screening, Station 3: Nutrition – healthy eating and sodium reduction, and Station 4: Exercise*” (HCW1) “*It should be a small group, about 10 people. They focus more and pay closer attention, possibly because they fear being asked questions. The session should be held on weekends, especially Sundays, when more people can attend*” (HCW2) *“I’d like to receive rewards as motivation for something useful in daily life. Some people may be unwilling to participate if they don’t receive anything in return*” (P3)
2. A blood pressure monitoring system for individuals with prehypertension.	1. All participants in the intervention group (n = 10) used the BP Wellness Kit. 2. The BP Wellness Kit was used by 30 participants over a 6-month period.	1. The appropriateness of the BP wellness kit	*“The equipment provided is appropriate and can be used effectively by patients. The record book is well-suited for older adults; unlike smaller notebooks that can be hard to read, the A4-sized format with large print used in this study enhances readability for the elderly”* (HCW2) *“Yes, it’s helpful and should continue to be available. The handbook is engaging and colourful”* (HCW1) *“It’s good as it is. The font size is fine. No need to add anything. It’s easy to understand”* (P10)
		2. Recommendations for the next phase of development	*“It may be more practical to replace the box with a cloth bag containing the logbook and educational materials, as the box is big and does not fit easily in motorbike baskets, making it difficult to carry”* (HCW1) “*If possible, it would be better to have more devices. Since patients need to use them for seven days, we must manage their use based on the available resources*” (HCW2)
3. Public relations through local loudspeakers and social media to raise awareness	1. All participants (n = 10) and community members received brief messages about hypertension prevention through local loudspeakers. 2. All participants (n = 10) and one family member each received information about hypertension prevention via the LINE application.	1. The village broadcast tower serves as an effective communication tool in rural communities	*“It was easy to listen to. The sound was clear and the audio system was good”* (P1) *“Morning is best since people are still at home. I listen while cooking and get health info at the same time. In the evening, people return home at different times”* (P2) *“The response (from people) has been excellent”* (HCW1)
		2. Addressing the challenges of using village broadcast tower for effective knowledge dissemination	*“I haven’t had time to prepare content due to a busy schedule. So, I’ve spoken with the public health officers, and we plan to restart this initiative”* (HCW1) *“Some of the content is useful and can be applied by patients. However, technical or academic content can be hard to understand — for example, information about sodium intake”* (HCW2)
		3. Social media posts motivated change	“*It’s good! Very useful. I read it every time. The visuals and instructions are clear and easy to understand. I can read it anytime”* (P3) *“Yes. For example, a post about cutting down on sugar, fat, and salt helped me rethink my food choices. I was about to eat something sweet, but after reading it, I decided to skip it (laughs)”* (P2)
		4. Limitations of using the LINE application	*“It’s suitable for some groups — those who have smartphones. But older adults who have vision problems or don’t use smartphones would face limitations”* (HCW2) “*It’s suitable for those with access — those who own smartphones and know how to use the LINE app. It’s not suitable for those unfamiliar with the technology*” (HCW1)
		5. Recommendations for the next phase of development	*“For those who can use it, it might be more engaging if presented as a cartoon or pop-up format”* (HCW1)

Abbreviations: HCW, health care worker; P, patient.

The broadcast tower served as an effective communication channel in rural communities. Participants reported that the messages were clear and could be heard while they were engaged in daily activities. One participant stated that the messages motivated her to avoid using high-sodium condiments. Social media posts delivered via the LINE application were acceptable and facilitated behavior change. Nevertheless, participants noted limited accessibility for older adults and those without smartphones.

Satisfaction with the community-based education on hypertension is presented in Supplemental Table S3 and Figure S2. Participants rated their knowledge and understanding of the information and intervention delivery at an excellent level, with mean scores of 4.73 and 4.53, respectively. The application of knowledge and trainer performance were rated at a good level overall with mean scores of 4.45 and 4.44, respectively. However, 14% of participants rated their trainer’s performance as neutral/fair.

### Blood Pressure Control

Blood pressure was measured at baseline, weekly for 4 weeks, and at the 6-week follow-up. The results showed a reduction in both systolic and diastolic blood pressure between the pre- and post-intervention periods. There was a decrease in systolic (129.3 mmHg to 123.9 mmHg; *P* = .004, n^2^p = 0.43), and diastolic blood pressure (83.9 mmHg to 79.9 mmHg; *P* < .001, n^2^p = 0.41), between baseline and the end of follow-up (Table 3). The mean reduction in systolic blood pressure between baseline and follow-up was 5.5 mmHg, and the mean reduction in diastolic pressure was 4.0 mmHg (Supplemental Figure S3).

**Table 3.** One Way Repeated Measures ANOVA Results on Blood Pressure, Waist Circumference, and Body Mass Index (N = 10).

Variable	Week (Mean, SD)						df	MS	F	*P* value	Partial eta squared (η^2^_p_)
	1Baseline	2	3	4	5	6Follow-up					
SBP(mmHg)	129.30(3.34)	124.19(5.85)	123.07(6.65)	123.29(5.50)	124.09(5.81)	123.90(5.04)	2.28	118.55	6.765^a^	.004	0.43
DBP(mmHg)	83.90(4.28)	80.96(2.03)	79.50(3.04)	78.99(3.06)	79.31(2.35)	79.90(2.07)	5	33.56	6.180^b^	<.001	0.41
WC(cm)	85.00(14.37)	84.90(14.45)	84.90(14.45)	84.80(14.35)	84.90(14.34)	84.60(14.37)	2.56	0.37	1.639^a^	.212	0.15
BMI(kg/m^2^)	26.52(7.85)	26.55(7.69)	26.53(7.70)	26.40(7.80)	26.43(7.88)	26.41(7.84)	1.53	0.15	0.593^a^	.523	0.06

Abbreviations: BMI, body mass index; DBP, diastolic blood pressure; df, degree of Freedom; MS, mean square; SBP, systolic blood pressure; WC, waist circumference.

a Greenhouse-Geisser correction was used to reduce type I error.

b Sphericity Assumed was applied.

### Change in Participant Knowledge, Behavior and Health Literacy

A paired *t*-test was conducted to evaluate the effect of the health education program on participants’ knowledge of hypertension and related health behaviors to improve blood pressure control. Participants’ mean knowledge score increased from 6.8 (pre-test) to 9.3 (post-test) (*t* = 9.3, *P* < .001, Hedges’*g* = 2.65). In addition, mean health literacy scores measured by the Thai HLHBQ increased by 10 points (SD = 5.97; *t* = 3.67, *P* = .005, Hedges’ *g* = 1.05). While there were observed changes in health behaviors, including dietary habits and physical activity, these changes were not statistically significant (*P* = .281 and *P* = .132, respectively). Health literacy, as measured by the ISHA-Q, showed improvement between baseline and follow-up (median score at baseline 584.5 to follow-up 617.0; Z = 2.09, *P* = .037, *r* = 0.66; Table 4).

**Table 4.** Comparison of Results on Knowledge, Health Literacy and Health Behaviors (N = 10).

Variable	Pre-test mean (SD) Median (IQR)	Post-test mean (SD) /Median (IQR)	Test statistic	*P*	Effect size (Hedges’g/r)
Knowledge	6.8 (0.92)	9.3 (0.67)	t(9) = 9.30	<.001***	2.65
Health literacy and Health behaviors by Thai HLHBQ					
Health literacy	63 (10.62)	73 (4.65)	t(9) = 3.67	.005**	1.05
Dietary behavior	19.9 (5.44)	21.70 (3.09)	t(9) = 1.73	.118	0.49
Exercise	7.2 (1.93)	7.9 (1.59)	t(9) = 1.66	.132	0.47
Health literacy by ISHA-Q	584.50 (237.25)	617.00 (57.00)	Z=2.09	.037*	0.66

Abbreviations: Thai HLHBQ, Thai Hypertensive Health Literacy and Health Behaviors Questionnaire; ISHA-Q, Information and Support for Health Action Questionnaire.

Wilcoxon signed-rank test was used for non-normally distributed variables. Pair *t*-test was used for normal distribution. Effect sizes reported as *r* for Wilcoxon and Hedges’ *g* for *t*-test.

* *P* < .05. **P < .01. ***P < .001.

### Waist Circumference and Body Mass Index

Among the 10 participants, there was a non-significant decrease in waist circumference from 85.00 to 84.60 (*P* = .212). In addition, although mean body mass index decreased from 26.52 to 26.41, this change was not statistically significant (*P* = .523; see Table 3).

## Discussion

This study aimed to evaluate the feasibility and acceptability of a community-based HeLP-PH, focusing on improving health literacy and blood pressure control. We found that the HeLP-PH program was both feasible and acceptable, as demonstrated by adherence to blood pressure monitoring at home, attendance at community-based education sessions, and retention in the study. There was also preliminary intervention evidence of effectiveness demonstrated by a reduction in blood pressure and an increase in health literacy at the 6-week follow-up visit. In addition, knowledge of hypertension and health behaviors improved immediately following the educational component of the program. No significant changes, however, were observed in uptake of health-promoting behaviors, waist circumference, or body mass index, possibly due to time limitations and a small sample size.

It seems that each participant responded differently due to varying biological, socioeconomic, and behavioral factors.^14^ For example, we found that half of the participants increased only their uptake of healthy dietary recommendations and experienced a decrease in systolic blood pressure of 3 to 11 mmHg, while 3 participants who improved their exercise participation scores showed a reduction of 3 to 8 mmHg. In addition, 1 participant who improved both diet and exercise behaviors had a decrease in systolic blood pressure of 8 mmHg. Only 1 participant, who showed no change in uptake of health-promoting behaviors, experienced an increase in systolic blood pressure. These results suggest that a culturally tailored intervention combined with community participation is feasible and acceptable and may have the potential to enhance health literacy and support blood pressure control.

Engaging multiple stakeholders through a co-design approach presents a promising strategy for setting priorities and formulating solutions and is increasingly recognized as an effective way to address health issues and achieve more sustainable outcomes, particularly in the management of noncommunicable diseases within primary health care.^6,25^ Community nurses should collaborate with multidisciplinary teams and engage community leaders, family members, and peer groups to support and motivate individuals with prehypertension, as such efforts may help bridge the intention–behavior gap and promote sustainable changes in health behaviors to prevent hypertension.^26^

The HeLP-PH program consisted of 3 interventions: a community-based educational project on hypertension, a blood pressure monitoring system, and public outreach through local loudspeakers and social media. Health education is essential not only to improve knowledge of health conditions and address varying levels of health literacy but also for controlling blood pressure.^1,8,9,18,19,27-29^ The community-based educational program was considered acceptable in terms of its materials, delivery methods, time, and setting. Participants preferred a half-day session because it was more convenient, reduced information overload, and was easier to attend. They also reported that the meeting room at the primary health care center was easily accessible. Multiple tools and materials can be incorporated into educational sessions, such as handbooks, videos for presentations, delivering content through human intermediaries, and cooking demonstrations using herbs to reduce sodium intake. These methods encouraged participants to enjoy the sessions and apply the information to their everyday lives. This aligns with a report by Wang et al,^30^ which illustrates that using diverse modes of delivering health information, such as lectures, handbooks, and videos, enhances the effectiveness of health education interventions and improves accessibility. Moreover, the educators or speakers were members of the community, which created a sense of belonging among the participants, as opposed to having outsiders lead the sessions. This finding is consistent with previous reports of randomized controlled trials on the effectiveness of health literacy interventions, which found that mean health literacy and health behavior scores increased, and blood pressure decreased after a 12-week intervention.^31^ Similarly, results from a systematic review reported that health education for individuals with prehypertension or hypertension in Southeast Asia significantly reduced systolic blood pressure by 5 to 18 mmHg over a period of 10 to 12 weeks.^19^ However, in this study the knowledge test was administered immediately before and after the teaching session and therefore primarily reflects short-term recall rather than durable learning. Several suggestions should be considered for improvements in the current co-designed intervention. Conventional teaching methods could be modified to promote more active learning, such as setting up learning stations with small groups of approximately 10 participants per station. Using dietary models when providing examples can help clarify the content. Additionally, offering gifts or souvenirs to participants may enhance motivation and engagement.

Home blood pressure monitoring over a 7-day period in individuals with prehypertension and the BP wellness kit was found to be practical and satisfactory, particularly the information handbook for hypertension prevention, which provided step-by-step guidance on how to measure blood pressure, getting to healthy behaviors, and used images rather than text. This was a novel approach, as the community had not previously been provided with a handbook featuring large fonts, clear illustrations, and colorful design elements. A prior feasibility study suggests that the informational materials should be improved to better suit participants, for example, by increasing the font size, refining content to be more tailored to the target audience, and incorporating more images and illustrations to enhance understanding.^28^

According to the 2024 Thai guidelines on hypertension, individuals with prehypertension are advised to measure their blood pressure at home every 7 days, both in the morning and before bedtime. Measuring blood pressure twice, 1 minute apart, provides more accurate data for the diagnosis of hypertension.^16^ However, 2 participants did not record their blood pressure readings at the scheduled times and instead measured them 2 to 3 hours later, after waking up or before going to bed, due to forgetfulness, rushing to work, or being too busy. Consequently, some blood pressure measurements may not accurately reflect the intended assessment times. In future implementation studies, participants should receive twice-daily reminders via the LINE application and be encouraged to involve family members in reminding them to improve adherence to the blood pressure monitoring schedule. Moreover, future studies should place greater emphasis on the timing of blood pressure measurement and consider providing either printed or electronic information handbooks to individuals with prehypertension who need to monitor their blood pressure at home.

Public relations aim to encourage participants to adopt healthy behaviors, reinforce the maintenance of those behaviors, and discourage or prevent unhealthy practices that may negatively affect health. Public relations through local broadcasting is an effective way to deliver and announce information appropriate for people living in the community, as it is a method commonly used for community-level communication in Thailand. This approach is consistent with previous findings, which show that education delivered through community broadcasting towers fosters a sense of participation and ownership among Thai residents, leading to increased public awareness and eagerness to learn and engage in activities.^32^

In addition, as we are currently living in the digital era, most Thai residents use digital platforms for communication, such as the LINE application and Facebook.^33^ This aligns with a previous study by Bawornthip et al,^21^ which reported that 36.1% and 32.1% of individuals with prehypertension in rural communities received health information through social media (Line and Facebook) and broadcasting towers, respectively. Participants accessed and read the posts whenever and wherever they needed health information. It appears that providing health information through social media can enhance access to health knowledge and services for individuals with prehypertension. To ensure the long-term sustainability of public relations, further efforts are required. Researchers in both broadcast media and social media should support healthcare providers by planning content in lay language, updating knowledge, and providing technology training.

### Strengths and Limitations

This is one of the first studies to use a rigorous convergent mixed-methods design to evaluate the feasibility and acceptability of a community-based health literacy program for individuals with prehypertension in Thailand. However, there are some limitations. First, this was a single-arm study without a control group. Second, the small sample size limits the ability to interpret the effectiveness of the intervention. Third, because participants were selected based on elevated blood pressure, the observed reductions cannot be distinguished from regression to the mean or white-coat effects. Fourth, the outcome measures were assessed only in the short term (6 weeks). A randomized controlled trial with long-term follow-up and increased sample size should be conducted in future research. Finally, the use of self-reported outcomes may introduce bias.

### Implications for Nursing Practice

Community nurses, healthcare providers, and community stakeholders play an essential role in delivering and supporting community-based health literacy programs. This study demonstrates that a health promotion education program to prevent hypertension is feasible, acceptable, and preliminary data in improving both blood pressure and health literacy among individuals with prehypertension in a community setting. Promoting health education, increasing health awareness through local pathways and the Line application, and providing equipment such as blood pressure monitors and health handbooks are necessary to support the population in changing behaviors to prevent hypertension. Implementing such an approach may help reduce long-term cardiovascular risk and promote sustainable health behavior change in similar rural communities.

## Conclusion

The results showed that the intervention was feasible and acceptable for implementation in a rural Thai community. Outcomes, including health literacy, knowledge, and blood pressure, were improved. Future studies should employ randomized controlled trials with larger sample sizes and focus on enhancing participants’ motivation and intention to change behavior.

## Supplemental Material

sj-pdf-1-wjn-10.1177_01939459261469267 – Supplemental material for Feasibility and Acceptability of a Community-Based Health Literacy Program for Individuals With Prehypertension Supplemental material, sj-pdf-1-wjn-10.1177_01939459261469267 for Feasibility and Acceptability of a Community-Based Health Literacy Program for Individuals With Prehypertension by Pataporn Bawornthip, Jo Mcdonall, Decha Tamdee, Andrea Driscoll and Anastasia Hutchinson in Western Journal of Nursing Research
