# Supplementary material for: Feasibility and Acceptability of a Community-Based Health Literacy Program for Individuals With Prehypertension
Source: West J Nurs Res. 2026 Aug 1;48(10):1115–24. doi: 10.1177/01939459261469267 (PMC13570976; doi:10.1177/01939459261469267)
Supplement: Supplementary file 1 [file sj-pdf-1-wjn-10.1177_01939459261469267.pdf]

Supplementary Table S1 The community-based Health Literacy Program for individuals with Prehypertension (HeLP-PH)

Week	Intervention	Objective	Activities
0		To collect data before start HeLP-PH	Conduct pre-test (HL, HB), measure BP, WC, BW
1	Public relations activities via a local broadcasting tower	1. To maintain continuous public relations through the local broadcasting tower and LINE application.<br>2. To increase knowledge of hypertension and how to prevent hypertension<br>3. To gain self-awareness<br>4. To increase motivation to change behavior	1. Participants and their communities received short messages related to hypertension prevention—such as the causes and effects of hypertension, and how to prevent it through exercise and a healthy diet—broadcast via the local broadcasting tower by a nurse practitioner.<br>2. Participants received infographic related to hypertension prevention, book how to cook low sodium, and video clip to demonstrate home exercise.
2		1. To increase knowledge of hypertension, healthy diet and exercise for preventing hypertension<br>2. To increase motivation to change behavior<br>3. To promote applying dietary information to practice<br>4. To increase self-awareness	Participants received education on:<br>1. Hypertension (causes, risk factors, complications)<br>2. Dietary behaviour (DASH diet; using a low-sodium model to show the sodium content of each condiment)<br>3. Exercise behavior (FITT principle; demonstration of walking using the 2:1:3 method or arm swings)<br>4. Reliable sources of health information relevant to hypertension prevention<br>5. Encouragement to use the Ask Me 3 technique<br>6. Motivation through planning necessary activities<br>7. Cooking demonstrations and processing local herbs as alternatives to condiments by community leader<br>8. Inviting individuals who have experienced stroke to share their symptoms and exchange knowledge<br>9. Conducted a pre-post evaluation of participants' knowledge and behaviors related to hypertension
	Public relations activities via the Line application	1. To increase knowledge of hypertension and how to prevent hypertension<br>2. To gain self-awareness	Participants were taught how to use the application LINE OA (Lod Seing Leang Rok) for giving health information 6 pillars:<br>1. Hypertension<br>2. The meaning of health numbers<br>3. Instruction to measure home blood pressure measurement

Week	Intervention	Objective	Activities
		3. To increase motivation to change behavior	4. Hypertension eBook and online blood pressure record<br>5. Dietary for prehypertension<br>6. Exercise for prehypertension
	A blood pressure monitoring system	1. To develop accurate knowledge of home blood pressure monitoring<br>2. To gain access to a blood pressure monitor and relevant health information<br>3. To regularly monitor blood pressure	Participants received the "BP Wellness Kit" and monitored their blood pressure twice daily, in the morning and evening, during weeks 2 to 5.
3	Public relations activities via a local broadcasting tower	1. To increase knowledge of hypertension and how to prevent hypertension<br>2. To gain self-awareness<br>3. To increase motivation to change behavior	Participants and their communities received short messages related to hypertension prevention—such as advice on healthy eating and the use of low-sodium condiments—broadcast via the local broadcasting tower by a nurse practitioner.
	Public relations activities via the Line application	1. To increase knowledge of hypertension and how to prevent hypertension<br>2. To gain self-awareness<br>3. To increase motivation to change behavior	Participants received infographics and videos demonstrating home exercises, the effects of uncontrolled blood pressure, methods for blood pressure control, and guidance on a healthy diet.
		1. To gain self-awareness<br>2. To increase motivation to change behavior	Participants were contacted via the Line application or by phone to follow up on their progress, provide encouragement, and offer advice on any issues related to behavior change.
4	Public relations activities via the Line application	1. To increase knowledge of hypertension and how to prevent hypertension<br>2. To gain self-awareness<br>3. To increase motivation to change behavior	Participants received infographics and videos demonstrating home exercises, the effects of uncontrolled blood pressure, methods for blood pressure control, and guidance on a healthy diet.
5	Public relations activities via a local broadcasting tower	1. To increase knowledge of hypertension and how to prevent hypertension<br>2. To gain self-awareness	Participants and their communities received short messages related to hypertension prevention—such as advice on exercise and dietary control—broadcast via the local broadcasting tower by a nurse practitioner.

Week	Intervention	Objective	Activities
		3. To increase motivation to change behavior	
	Public relations activities via the Line application	1. To increase knowledge of hypertension and how to prevent hypertension<br>2. To gain self-awareness<br>3. To increase motivation to change behavior	Participants received infographics and videos demonstrating home exercises, the effects of uncontrolled blood pressure, methods for blood pressure control, and guidance on a healthy diet.
6		To collect data after finish HeLP-PH	- Conducted post-test (HL, HB), measure BP, WC, BW<br>- Conducted focus group, semi-structured interviews, satisfaction survey to evaluate feasibility, usability, and acceptability

Noted: BP: blood pressure, HL: health literacy, HB: health behaviour, WC: waist circumference, BMI: body mass index, DASH: dietary approaches to stop hypertension, FITT: frequency, intensity, time, and type of exercise, LINE OA: LINE official account

Supplementary Figure S1 The three projects in the community-based Health Literacy Program for individuals with Prehypertension (HeLP-PH)

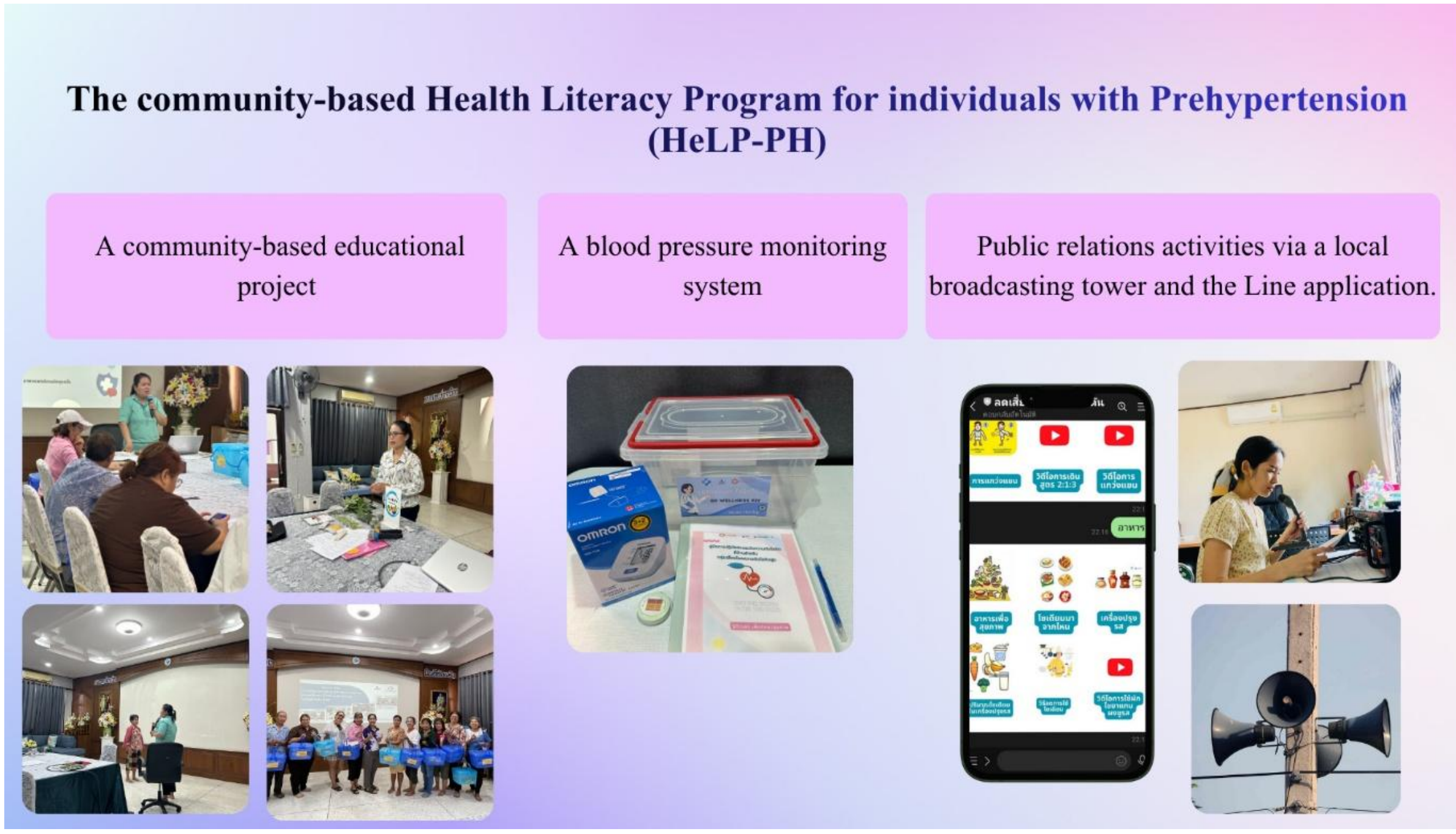

Supplementary Table S2 Measurement model result

Global fit and misfit indices were reported, including the χ^2/df ratio, Comparative Fit Index (CFI), Tucker-Lewis Index (TLI), and the Root Mean Square Error of Approximation (RMSEA). The results from the confirmatory factor analysis (CFA) demonstrated an adequate fit of the measurement model to the data ($\chi^2 = 4753.25$, $p < .001$; $\chi^2/df = 2.34$; RMSEA = 0.07, $p < .001$; CFI = 0.76; TLI = 0.73).

Variables	Items	Factor loading	Standard error (SE)	Average variance extracted (AVE)	Composited reliability (CR)	Cronbach's alpha
1. Knowledge about service entitlements	I35	0.77	0.60	0.565	0.838	0.840
	I29	0.77	0.45			
	I21	0.75	0.09			
	I45	0.72	0.06			
2. Awareness of resources that support health in your neighbourhood	I46	0.78	0.05	0.558	0.863	0.858
	I39	0.81	0.03			
	I52	0.77	0.04			
	I32	0.66	0.05			
	I48	0.72	0.06			
3. Ability to access health services	I53	0.83	0.03	0.537	0.851	0.838
	I62	0.80	0.05			
	I5	0.59	0.06			
	I41	0.74	0.05			
	I66	0.68	0.08			
4. Ability to get the information and advice you want from health professionals	I1	0.61	0.06	0.580	0845	0.834
	I25	0.84	0.04			
	I64	0.77	0.05			
	I34	0.80	0.03			
5. Close support people	I63	0.79	0.05	0.603	0.857	0.844
	I27	0.64	0.06			
	I38	0.84	0.04			
	I56	0.85	0.03			
6. Ability to find suitable health information	I13	0.74	0.06	0.614	0.863	0.859
	I44	0.72	0.06			
	I59	0.83	0.04			
	I15	0.84	0.05			
	I19	0.73	0.05	0.635	0.874	00.873
	I6	0.79	0.04			

Variables	Items	Factor loading	Standard error (SE)	Average variance extracted (AVE)	Composited reliability (CR)	Cronbach's alpha
7. Evaluating the trustworthiness of health information	I11	0.84	0.03			
	I57	0.82	0.04			
8. Accepting responsibility for health	I7	0.66	0.06	0.493	0.795	0.796
	I36	0.73	0.06			
	I18	0.71	0.07			
	I14	0.70	0.06			
9. Eating for health	I31	0.74	0.05	0.495	0.795	0.793
	I2	0.63	0.06			
	I43	0.80	0.04			
	I9	0.63	0.05			
10. Exercise for health	I22	0.63	0.07	0.531	0.817	0.825
	I42	0.71	0.05			
	I12	0.71	0.07			
	I58	0.84	0.03			
11. Managing stress	I50	0.69	0.06	0.552	0.831	0.83
	I33	0.77	0.03			
	I54	0.72	0.05			
	I17	0.80	0.04			
12. Travelling barriers and ability	I4	0.76	0.05	0.594	0.853	0.851
	I20	0.83	0.03			
	I16	0.78	0.04			
	I10	0.71	0.05			
13. Using medicines	I40	0.81	0.08	0.583	0.874	0.875
	I65	0.77	0.10			
	I8	0.70	0.09			
	I24	0.71	0.09			
	I55	0.82	0.04			
14. Using herbs and supplements	I61	0.78	0.04	0.673	0.891	0.888
	I47	0.88	0.03			
	I37	0.82	0.05			
	I28	0.80	0.04			
15. Exchange experience and knowledge with other patients	I67	0.78	0.05	0.525	0.814	0.819
	I23	0.65	0.05			
	I30	0.79	0.04			
	I51	0.67	0.07			
	I3	0.62	0.05	0.463	0.773	0.762

Variables	Items	Factor loading	Standard error (SE)	Average variance extracted (AVE)	Composited reliability (CR)	Cronbach's alpha
16. Self-monitoring	I49	0.60	0.08			
	I26	0.69	0.05			
	I60	0.80	0.03			

Note: All standard factor loading are significant at p<.001

Supplementary Table S3 Satisfaction assessment form

Items	Average score
1. Trainer Performance	
1.1 The trainer demonstrated clarity in delivering the content.	4.4
1.2 The trainer explained the subject matter accurately and concisely.	4.6
1.3 Opportunities were provided for participants to exchange ideas and share experiences.	4.3
1.4 The trainer responded to participants' questions effectively.	4.4
1.5 The training materials provided were relevant and well-prepared.	4.5
2. Knowledge and understanding	
2.1 Participants' understanding of the topic improved following the training.	4.6
2.2 Participants were able to explain the content in detail.	4.8
2.3 Participants were able to identify and articulate the benefits and advantages of the topic.	4.8
3. Application of Knowledge	
3.1 Participants were able to apply the acquired knowledge to their professional practice.	4.8
3.2 Participants were able to disseminate the knowledge to peers and family members.	4.6
3.3 Participants were able to provide advice based on the knowledge gained.	4.1
3.4 Participants expressed confidence in utilizing the knowledge in real-life contexts.	4.3
4. Intervention delivery	
4.1 The format and venue of the project were appropriate and conducive to learning.	4.4
4.2 Training materials and supporting media were adequate and useful.	4.6
4.3 Audio-visual equipment was sufficiently prepared and functional.	4.7
4.4 The duration and schedule of the project were appropriate.	4.5
4.5 Coordination among stakeholders was effective and efficient.	4.3
4.6 Services and logistical support provided during the project were satisfactory.	4.7

Criteria

4.51- 5.00	Exellent
3.51 - 4.50	Good
2.51 - 3.50	Neutral
1.51 - 2.50	Fair
1.00 - 1.50	Improve

Supplementary Figure S2 The satisfaction of a community-based educational project (n=10)

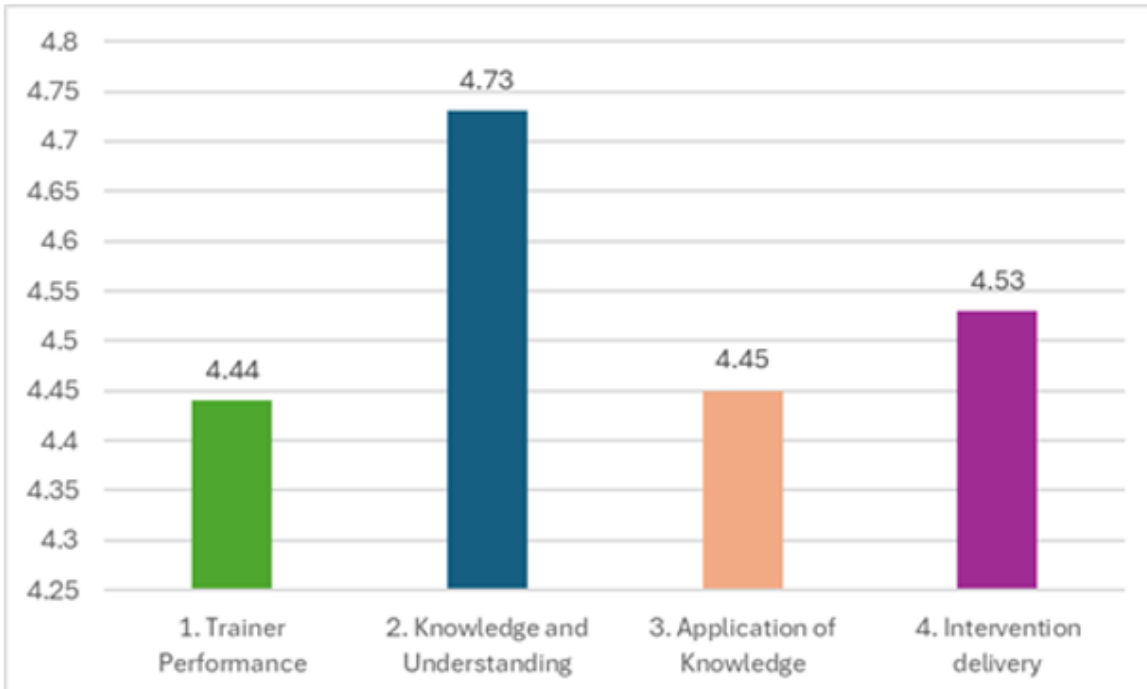

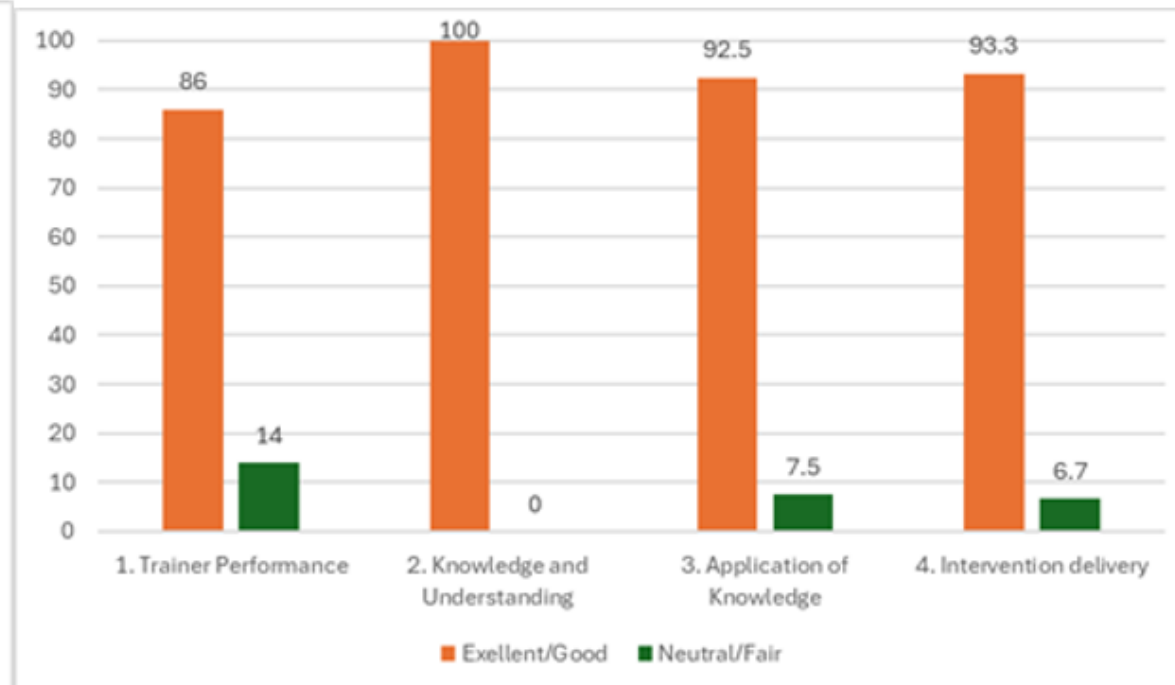

a. The mean satisfaction score for each component

b. The proportion of responses rates as Exellent/Good versus Neutral/Fair

Supplementary Figure S3 The mean difference in blood pressure between week 1 and week 6 (N=10)

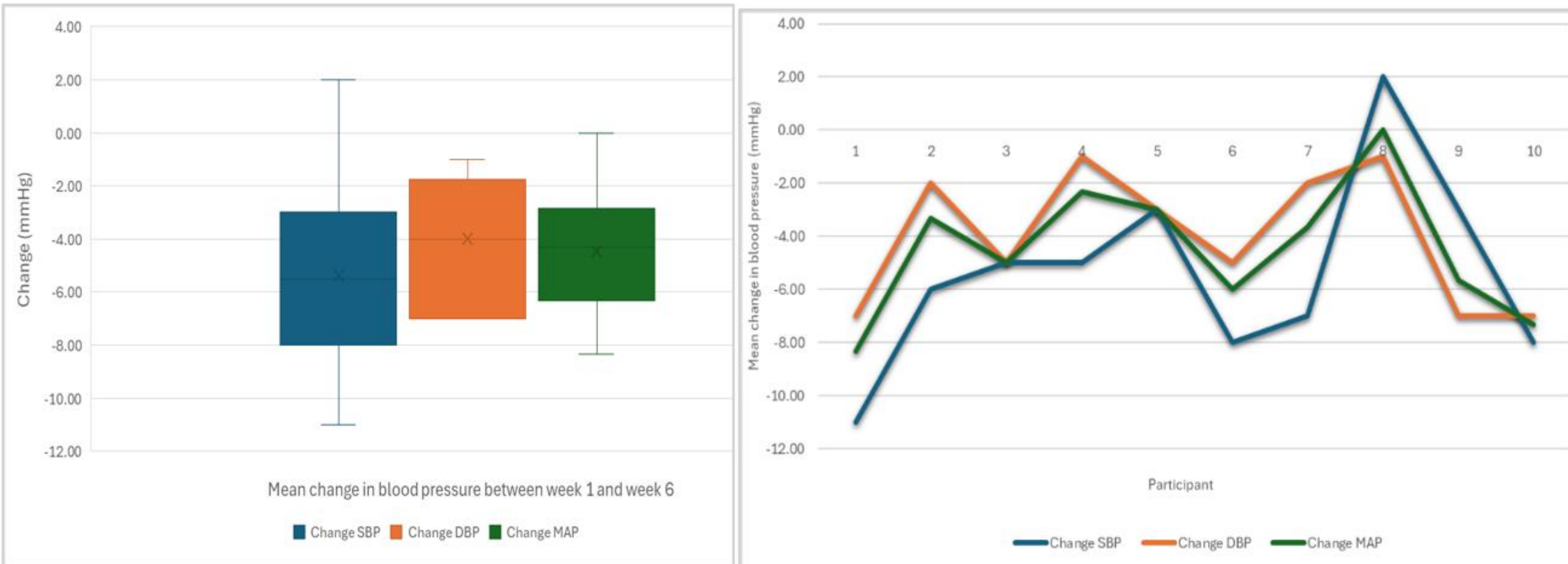

a. mean change between group

b. mean change within individual
